# Low‐density lipoprotein receptor is required for cholesteryl ester transfer protein to regulate triglyceride metabolism in both male and female mice

**DOI:** 10.14814/phy2.14732

**Published:** 2021-02-24

**Authors:** Brian T. Palmisano, Sophia Yu, Joshua C. Neuman, Lin Zhu, Thao Luu, John M. Stafford

**Affiliations:** ^1^ Tennessee Valley Health System Veterans Affairs Nashville TN USA; ^2^ Department of Molecular Physiology & Biophysics Vanderbilt University School of Medicine Nashville TN USA; ^3^ Division of Cardiovascular Medicine Stanford University Medical Center Stanford CA USA; ^4^ Department of Medicine Division of Diabetes, Endocrinology and Metabolism Vanderbilt University Medical Center Nashville TN USA

**Keywords:** cholesteryl ester transfer protein (CETP), low‐density lipoprotein receptor (LDLR), sex differences, triglyceride (TG)

## Abstract

Elevated triglycerides (TGs) and impaired TG clearance increase the risk of cardiovascular disease in both men and women, but molecular mechanisms remain poorly understood. Cholesteryl ester transfer protein (CETP) is a lipid shuttling protein known for its effects on high‐density lipoprotein cholesterol. Although mice lack CETP, transgenic expression of CETP in mice alters TG metabolism in males and females by sex‐specific mechanisms. A unifying mechanism explaining how CETP alters TG metabolism in both males and females remains unknown. Since low‐density lipoprotein receptor (LDLR) regulates both TG clearance and very low density lipoprotein (VLDL) production, LDLR may be involved in CETP‐mediated alterations in TG metabolism in both males and females. We hypothesize that LDLR is required for CETP to alter TG metabolism in both males and females. We used LDLR null mice with and without CETP to demonstrate that LDLR is required for CETP to raise plasma TGs and to impair TG clearance in males. We also demonstrate that LDLR is required for CETP to increase TG production and to increase the expression and activity of VLDL synthesis targets in response to estrogen. Additionally, we show that LDLR is required for CETP to enhance β‐oxidation. These studies support that LDLR is required for CETP to regulate TG metabolism in both males and females.

## Graphical abstract

Transgenic CETP expression in mice raises TGs in both males and females, but through distinct mechanisms. LDLR is required for CETP to impair TG clearance in males. In females, LDLR is required to raise TG production in response to estrogen and is required to enhance beta‐oxidation. LDLR represents a unifying target through which CETP alters TG metabolism.

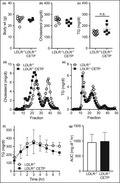

## 1 INTRODUCTION

Sex differences in triglyceride (TG) metabolism may explain some of the sex differences in the risk of cardiovascular disease. Hypertriglyceridemia is an important risk factor for cardiovascular disease (Hokanson & Austin, 1996; Langsted et al., 2008; Patel et al., 2004; Sprecher et al., 2000). Both overproduction and impaired clearance of plasma TGs contribute to elevated plasma TGs. While elevated fasting plasma TGs may more strongly affect risk for cardiovascular disease in women, impaired postprandial TG clearance increases the risk of cardiovascular disease in both men and women (Boquist et al., 1999; Carstensen et al., 2004; Freiberg et al., 2008; Ginsberg et al., 1995; Groot et al., 1991; Nordestgaard et al., 2007; Sharrett et al., 1995; Teno et al., 2000). Understanding the pathways that regulate sex differences in TG clearance may lead to novel therapies that reduce the risk of cardiovascular disease.

We previously demonstrated that transgenic expression of cholesteryl ester transfer protein (CETP) results in increased plasma TG levels in female and male mice, but by distinct mechanisms (Palmisano et al., 2016, 2020). Mice naturally lack CETP and subsequently carry most cholesterol in HDL. Transgenic expression of CETP in mice generates a lipid profile more similar to humans with lipid distributed in very low‐density lipoprotein (VLDL) and LDL as well as HDL (Pape et al., 1991). Therefore, transgenic expression of CETP in mice more closely models human lipid physiology. Furthermore, the use of mice with transgenic expression of CETP permits the study of novel functions of CETP without the potentially toxic or off‐target effects of CETP inhibitors (Barter et al., 2007). We previously found that transgenic expression of CETP alters sex hormone biology to impact TG metabolism in both male and female mice. In female mice, CETP expression increased TG production in response to estrogen (Palmisano et al., 2016). In males, CETP expression impaired postprandial TG clearance (Palmisano et al., 2020). In females, CETP signals *via* two distinct liver networks that govern separate aspects of TG metabolism (Palmisano et al., 2016). In males, CETP signals through the liver androgen receptor (Palmisano et al., 2020). It remains to be determined if CETP has a common molecular target regulating TG metabolism in both males and females.

Low‐density lipoprotein receptor (LDLR) has well‐known roles in lipid clearance, but also regulates VLDL assembly. LDLR is a cell surface transmembrane receptor that binds to apolipoprotein B (apoB) or apolipoprotein E (apoE) on chylomicrons, VLDL or LDL (Jeon & Blacklow, 2005). Upon lipoprotein binding to LDLR, holoparticle uptake *via* clathrin‐mediated endocytosis results in cellular uptake of lipoproteins (Brown & Goldstein, 1979). LDLR has a well‐established role in TG clearance (Bilheimer et al., 1979; Horton et al., 1999; Ishibashi et al., 1996; James et al., 1989; Kypreos & Zannis, 2006). In addition to regulating TG clearance, LDLR also regulates VLDL export *in vitro* (Horton et al., 1999; Twisk et al., 2000), in mouse models (Coenen et al., 2007; Millar et al., 2002; Teusink et al., 2001), and in humans with mutations in *LDLR* (Bilheimer et al., 1979; Cummings, 1995; James et al., 1989; Tremblay et al., 2004; Zulewski et al., 1998). Since LDLR is involved in both TG clearance and TG production, LDLR represents a unique molecular target that may mediate the effects of CETP on TG metabolism in both male and female mice.

Here, we determine whether LDLR plays a role in regulating TG metabolism in male and female mice expressing CETP. Because of the dual role of LDLR in both TG clearance and production, we hypothesized that LDLR was required for CETP to regulate TG clearance in males and TG production in females. We used male and female LDLR null mice with and without CETP to define if CETP expression alters TG metabolism in the absence of LDLR. In males, we demonstrate that LDLR is required for CETP to raise plasma TG levels and to impair TG clearance. In females, we demonstrate that LDLR is required for CETP to raise plasma TGs and to raise TG production in response to estrogen. Also, in females, we demonstrate that LDLR is required for CETP to enhance β‐oxidation. Thus, LDLR may be a mediator by which CETP regulates TG metabolism in both males and females. This study adds to a growing body of work suggesting that CETP has additional functions beyond its conventional role in HDL cholesterol metabolism.

## 2 MATERIALS AND METHODS

### 2.1 Animals

All mouse experiments were approved under the Vanderbilt University Institutional Animal Care and Use Committee. Mice were housed in 12 h light/dark cycles in temperature and humidity‐controlled facilities with ad‐libitum access to chow diet and water. Transgenic CETP mice were purchased from the Jackson Laboratories (C57BL/6‐Tg(CETP)UCTP20Pnu/J, Strain: 001929, Jackson Laboratories). We previously established the role of CETP on TG metabolism in both males and females relative to WT mice (Palmisano et al., 2016, 2020). LDLR deletion has been shown to impair TG clearance and increase TG production, contributing to elevated TGs relative to WT mice (Coenen et al., 2007; Ishibashi et al., 1996; Teusink et al., 2001). Here, we asked if CETP could raise plasma TGs in the absence of LDLR using LDLR null mice with and without CETP. CETP was bred onto mice with a global LDLR knockout (B6.129S7‐Ldlr^tm1Her^/J, Strain: 002207, backcrossed 13 generations onto C57BL/6J background; Jackson Laboratories). Once the LDLR knockout allele was bred to homozygosity, LDLR^−/−^ CETP^+/−^ and LDLR^−/−^ (naturally lacking CETP) littermates were used. Mice used in this study were aged 3–4 months old to ensure complete sexual development. All animals were euthanized between 8 and 11 am to minimize circadian variation in gene expression. All females were ovariectomized to remove the contribution of endogenous ovarian hormones, as described previously (Palmisano et al., 2016). After recovering 6–7 days, females were injected subcutaneously with vehicle (100 μl, sesame oil; Sigma) or estrogen (1 μg/g, β‐estradiol‐3‐benzoate, Sigma). Mice were sacrificed 24 hr after estrogen treatment to prevent changes associated with long‐term estrogen replacement, such as reduced adiposity, reduced insulin, and increased free fatty acids (D'Eon et al., 2005).

### 2.2 Genotyping

Mice were genotyped using tail DNA. The CETP transgene was detected in a multiplexed PCR reaction containing CETP‐F (GAATGTCTCAGAGGACCTCCC), CETP‐R (CTTGAACTCGTCTCCCATCAG), Control‐F (CTAGGCCACAGAATTGAAAGATCT), Control‐R (GTAGGTGGAAATTCTAGCATCATCC). The LDLR knockout allele was detected in a multiplexed PCR reaction using LDLR‐F (TATGCATCCCCAGTCTTTGG), LDLR‐WT‐R (CTACCCAACCAGCCCCTTAC), and NEO‐R (ATAGATTCGCCCTTGTGTCC).

### 2.3 Lipid and lipoprotein analysis

Blood was collected in EDTA‐containing tubes. Plasma TG and cholesterol were measured using colorimetric kits (Infinity, Sigma). Lipoproteins were separated using fast‐performance liquid chromatography (FPLC) on a Superose6 column (GE Healthcare) from 150 μl pooled plasma from all mice within the same experimental group. Liver TG content, liver cholesterol content, and plasma total testosterone levels were determined by the Vanderbilt Hormone Assay Core. For liver TAG and cholesterol content, 50–100 mg liver tissue was Folch extracted and separated by thin‐layer chromatography, which was then analyzed by gas chromatography with internal standards used to control for the efficiency of extraction. Plasma β‐hydroxybutyrate was measured from blood collected following an 18 hr overnight fast using a colorimetric kit (Cayman).

### 2.4 *In vivo* TG clearance and production

To measure TG clearance, 12‐h fasted mice were orally gavaged with olive oil (200 μl/mouse) and plasma TG was measured from tail blood sampling over 5–7 h. To measure TG production, 3‐h fasted mice were given 200 μl intraperitoneal Poloxamer‐407 (1000 mg/kg; Sigma), an inhibitor of lipolysis (Millar et al., 2005), and plasma TG was measured over 2 h.

### 2.5 Liver mRNA Expression

Liver samples for mRNA analysis were kept in RNA‐Later (Thermo) at 4°C overnight and then stored at −20°C. A small piece of liver tissue was bead homogenized in Trizol (Qiagen). RNA was isolated according to the manufacturer's instructions. Complementary DNA was synthesized from 1 µg of mRNA (iScript; Bio‐Rad). RT‐PCR was done in triplicate from 10 ng cDNA (JumpStart Taq ReadyMix; Sigma). Primers were validated using a melting curve and annealing temperatures were optimized using gradient RT‐PCR. Gene expression was quantified using efficiency corrected ΔCt method with normalization of genes to *Ppia* (Pfaffl, 2001). Primer efficiency was measured for each RT‐PCR reaction using LinRegPCR (Ramakers et al., 2003). Primers are listed in Table 1.

**TABLE 1:** RT‐PCR Primers

Gene	Forward primer	Reverse primer
*Acox1*	TAACTTCCTCACTCGAAGCCA	AGTTCCATGACCCATCTCTGTC
*Apob*	GCCCATTGTGGACAAGTTGATC	CCAGGACTTGGAGGTCTTGGA
*Arf1*	CTGGGCGAAATTGTGACCAC	TCCACTACGAAGATCAAGCCTT
*Cpt1a*	CTCAGTGGGAGCGACTCTTCA	GGCCTCTGTGGTACACGACAA
*Cpt2*	CAGTGCACAGAAGCCTCTCTTG	CTTCCCAATGCCGTTCTCAA
*Mttp*	CAAGCTCACGTACTCCACTGAAG	TCATCATCACCATCAGGATTCCT
*P4hb*	GCCGCAAAACTGAAGGCAG	GGTAGCCACGGACACCATAC
*Pdia3*	CGCCTCCGATGTGTTGGAA	CAGTGCAATCCACCTTTGCTAA
*Pdia4*	TCCCATTGCTGTAGCGAAGAT	GGGGTAGCCACTCACATCAAAT
*Ppara*	TATTCGGCTGAAGCTGGTGTAC	CTGGCATTTGTTCCGGTTCT
*Ppia*	CGATGACGAGCCCTTGG	TCTGCTGTCTTTGGAACTTTGTC
*Sort1*	TGTGGCCAAGCAGCCATCCG	TCAGGCTGCTCCACGCACTC

### 2.6 Statistical Analysis

All data are summarized using mean and SD. Statistical tests between two groups were analyzed by unpaired Student's *t*‐test. Data with more than one group were analyzed by one‐way ANOVA with Bonferroni post hoc comparisons of selected columns. Repeated measures one‐way ANOVA was used for measures of plasma TG over time with Bonferroni posttest comparisons. Genotype effects were determined by two‐way ANOVA. *p* < 0.05 were considered statistically significant.

## 3 RESULTS

### 3.1 CETP does not raise plasma TGs in males in the absence of LDLR

**FIGURE 1:**
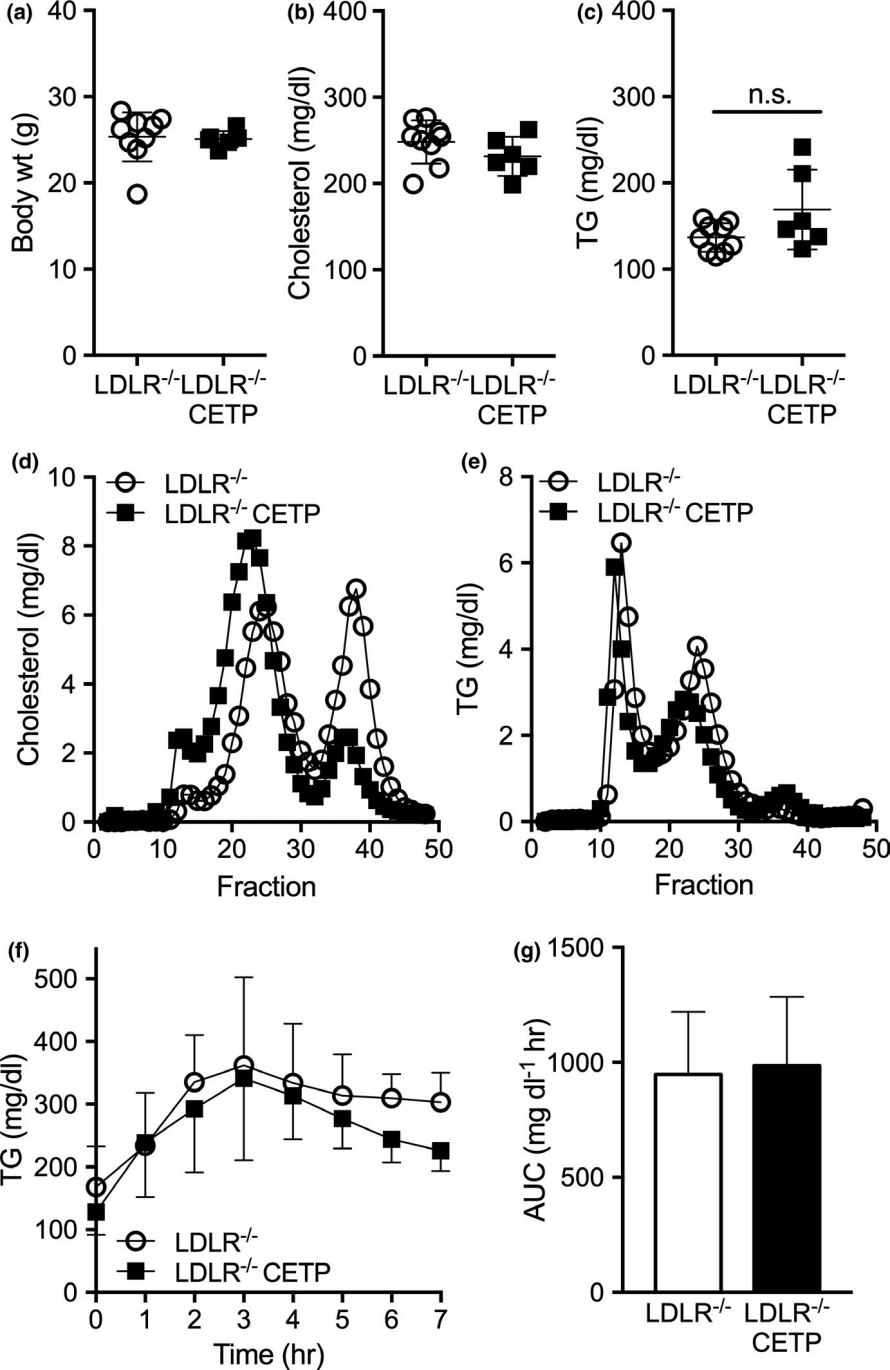
Effect of cholesteryl ester transfer protein (CETP) on plasma triglyceride (TG) metabolism in male mice lacking low‐density lipoprotein receptor (LDLR). CETP was crossed onto the LDLR^−/−^ as described in Section 2. Ad lib fed male LDLR^−/−^ CETP transgenic and LDLR^−/−^ littermates were sacrificed between 8 and 11 am. (a) Body weight of LDLR^−/−^ CETP and LDLR^−/−^ males (*n* = 6–9/group). (b, c). Plasma cholesterol (b) and plasma TGs (c) were of LDLR^−/−^ CETP and LDLR^−/−^ males (*n* = 6‐9/group). (d–e) Cholesterol content (d) and TG content (e) of lipoproteins in from FPLC separated pooled plasma of male LDLR^−/−^ CETP and LDLR^−/−^ males. (f–g) 12 h overnight fasted CETP LDLR^−/−^ CETP and LDLR^−/−^ littermates were gavaged with olive oil (200 μl/mouse) and postprandial plasma TGs were measured over 7 h. Postprandial TG levels (f) and AUC (g) in LDLR^−/−^ CETP and LDLR^−/−^ males (*n* = 8–9/group)

We recently demonstrated that CETP impairs postprandial TG clearance in males through liver androgen receptor signaling (Palmisano et al., 2020). Since LDLR is a major determinant of plasma TG clearance, LDLR may play a role in CETP‐mediated regulation of TG clearance. Here, we test the hypothesis that LDLR is required for CETP to impair postprandial TG clearance in males. To test this, we crossed CETP onto LDLR knockout mice to generate LDLR^−/−^ mice both with and without CETP. CETP expression did not alter body weight in LDLR^−/−^CETP males relative to LDLR^−/−^ littermates (Figure 1a). In the absence of LDLR, CETP did not alter plasma cholesterol levels (Figure 1b). Additionally, in the absence of LDLR, CETP did not raise plasma TG levels relative to LDLR^−/−^ littermate males (Figure 1c). CETP expression reduced HDL cholesterol content and increased VLDL and LDL cholesterol content, an expected effect of CETP‐mediated lipid transfer (Figure 1d). In the absence of LDLR, CETP did not significantly alter TG content of HDL, LDL, or VLDL (Figure 1e). CETP expression slightly increased lipoprotein size, as shown by the left‐ward shift in FPLC separated plasma lipoproteins in LDLR^−/−^CETP males relative to LDLR^−/−^ males (Figure 1d,e). In mice with normal LDLR expression we previously showed that CETP raises plasma TGs in male mice (Palmisano et al., 2020). Thus, this study supports that LDLR is required for CETP to raise plasma TG in males.

Since we previously showed that CETP raised plasma TGs by impairing postprandial TG clearance in males (Palmisano et al., 2020), we next sought to determine whether LDLR was required for CETP to impair postprandial TG clearance. In the absence of LDLR, CETP expression did not alter postprandial TG excursion in males (Figure 1f). TG clearance, as indicated by the area under the curve, was not impaired in LDLR^−/−^CETP males relative to LDLR^−/−^ males (Figure 1g). Thus, LDLR is required for CETP to impair postprandial TG clearance in males.

### 3.2 CETP does not raise plasma TGs in females in the absence of LDLR

**FIGURE 2:**
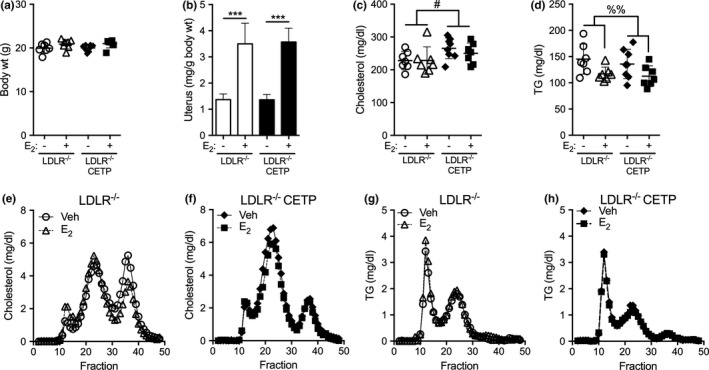
Effect of cholesteryl ester transfer protein (CETP) on estrogen regulation of plasma lipids in females lacking low‐density lipoprotein receptor (LDLR). Ad lib fed female LDLR^−/−^ CETP transgenic and LDLR^−/−^ littermates were given sesame oil (veh) or estradiol (E_2_, 1 mg/kg) and sacrificed 24 h later between 8 and 11 am. (a) Body weight was not different between LDLR^−/−^ CETP and LDLR^−/−^ females treated with veh or E_2_ (*n* = 7‐8/group). (b) Uterine mass increased similarly in LDLR^−/−^ CETP and LDLR^−/−^ females with E_2_ treatment (*n* = 7–8/group, ***p < 0.001, *t*‐test). (c, d) Plasma cholesterol (c) and plasma TG (d) in LDLR^−/−^ CETP and LDLR^−/−^ females treated with veh and E_2_. (^#^ *p* < 0.05 for genotype effect, ^%%^ *p* < 0.01 for E_2_ effect, two‐way ANOVA). (e–h) Cholesterol content (e, f) and TG content (g, h) of lipoproteins in from FPLC separated pooled plasma of female LDLR^−/−^ CETP (e, g) and LDLR^−/−^ (f, h) females treated with veh or E_2_

We previously demonstrated that expression of CETP increased plasma TGs and increased VLDL‐TG production in response to estrogen in females (Palmisano et al., 2016). Since LDLR was required for CETP to alter plasma TGs in males, we next sought to determine if LDLR was also required for CETP to enhance TG production in females in response to estrogen. Females were ovariectomized to reduce variability in estrus cycling and to remove the contribution of endogenous hormones. Body weight and uterine response to estrogen treatment were similar in both LDLR^−/−^ and LDLR^−/−^CETP females (Figure 2a,b). Estrogen treatment did not alter plasma cholesterol in LDLR^−/−^ and LDLR^−/−^CETP females (Figure 2c). CETP expression in LDLR^−/−^ females modestly increased plasma cholesterol levels (^#^ *p* < 0.05 for genotype effect, Figure 2c). In the absence of LDLR, CETP expression failed to raise plasma TGs in females (Figure 2d). Estrogen modestly reduced plasma TGs similarly in both LDLR^−/−^ and LDLR^−/−^CETP females (^%%^ *p* < 0.01 for estrogen effect, Figure 2d), but was not statistically significant in either group by post hoc testing. In both LDLR^−/−^ or LDLR^−/−^CETP females, TG and cholesterol content of pooled lipoproteins were qualitatively similar with estrogen treatment (Figure 2e–h). As expected and similar to males, CETP expression increased cholesterol content of LDL and reduced cholesterol content of HDL (Figure 2f), which is due to the lipid shuttling activity of CETP in plasma. Previously, estrogen treatment increased plasma TGs and nearly doubled the TG content of VLDL in CETP female mice (Palmisano et al., 2016). In the absence of LDLR, however, estrogen failed to increase TG content of VLDL in LDLR^−/−^CETP females (Figure 2h). Thus, LDLR is required for estrogen to raise plasma TGs and increase the TG content of VLDL in CETP females.

### 3.3 CETP does not raise TG production in response to estrogen in females without LDLR

**FIGURE 3:**
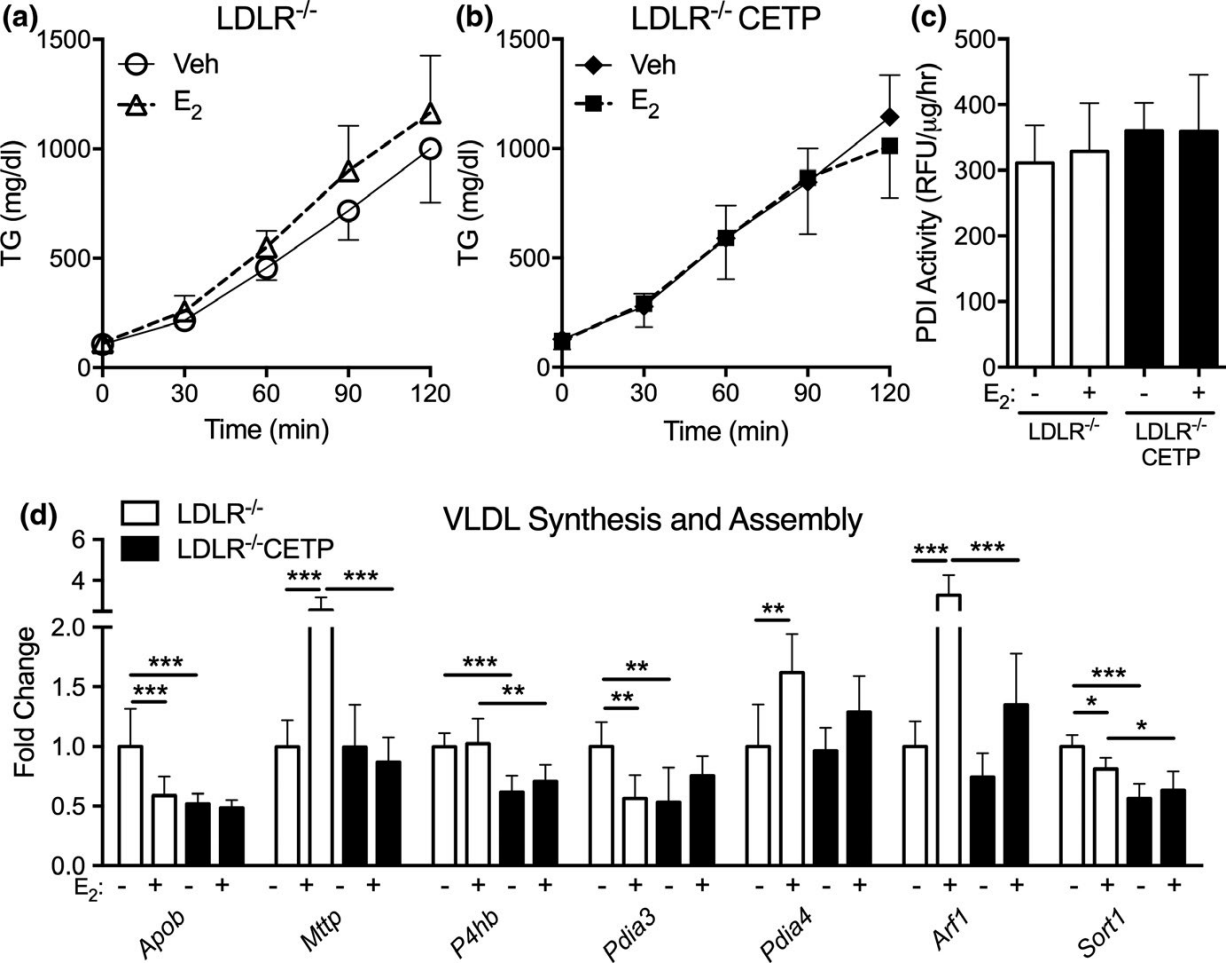
Effect of cholesteryl ester transfer protein (CETP) on estrogen regulation of triglyceride (TG) production and liver expression of VLDL target genes in females lacking low‐density lipoprotein receptor (LDLR). Female LDLR^−/−^ CETP and LDLR^−/−^ littermates were given sesame oil (veh) or estrogen (E_2_) 24 h prior to study. On the day of the study, mice were fasted 3 h and given poloxamer 407 (1 g/kg) and plasma TG were measured over 2 h. (a, b). Plasma TG production was measured over time after treatment with veh and E_2_ in LDLR^−/−^ (A) and LDLR^−/−^CETP females. (b) (*n* = 7‐8/group). (c) Liver protein disulfide isomerase (PDI) activity was measured in ad lib fed females treated with veh or E_2_ 24 h prior to sacrifice. (*n* = 7–8/group). (d) Liver mRNA levels of genes of VLDL synthesis and assembly in LDLR^−/−^ CETP and LDLR^−/−^ females treated with veh or E_2_ (*n* = 7–8/group, *p < 0.05, **p < 0.01, ***p < 0.001, one‐way ANOVA with post hoc comparison)

To confirm that LDLR is required for CETP to raise VLDL production in response to estrogen, we measured TG production in LDLR^−/−^ and LDLR^−/−^ CETP females following estrogen and vehicle treatment. Estrogen did not alter TG production in LDLR^−/−^ or LDLR^−/−^ CETP females (Figure 3a,b). We previously showed that CETP alters estrogen regulation of liver mRNA expression and protein activity of genes governing VLDL synthesis and assembly, especially expression and activity of protein disulfide isomerase (PDI; Palmisano et al., 2016). In the absence of LDLR, estrogen failed to increase liver PDI activity in LDLR^−/−^CETP females (Figure 3c). Furthermore, estrogen failed to increase expression of mRNA for PDI isoforms or other genes involved in VLDL synthesis and assembly in LDLR^−/−^CETP females (Figure 3d). Previously, estrogen did not significantly alter liver mRNA expression of genes involved in VLDL synthesis and assembly in WT females (Palmisano et al., 2016). In LDLR^−/−^ females, however, estrogen significantly altered expression of a number of genes in the VLDL synthesis and assembly pathway (*Apob*,*Mttp*,*Pdia3*,*Pdia4*,*Arf1*,*Sort1*, Figure 3d). Deletion of LDLR, therefore, seems to generate a novel response to estrogen on genes involved in VLDL synthesis and assembly. Since estrogen both increased and decreased expression of genes in the VLDL pathway, the net effect of estrogen did not alter VLDL‐TG production (Figure 3a). Overall, these results support that LDLR is required for CETP expression to increase TG production and expression and activity of genes involved in VLDL synthesis and assembly in response to estrogen.

### 3.4 LDLR is required for CETP to enhance liver β‐oxidation, but not lower liver TG content

**FIGURE 4:**
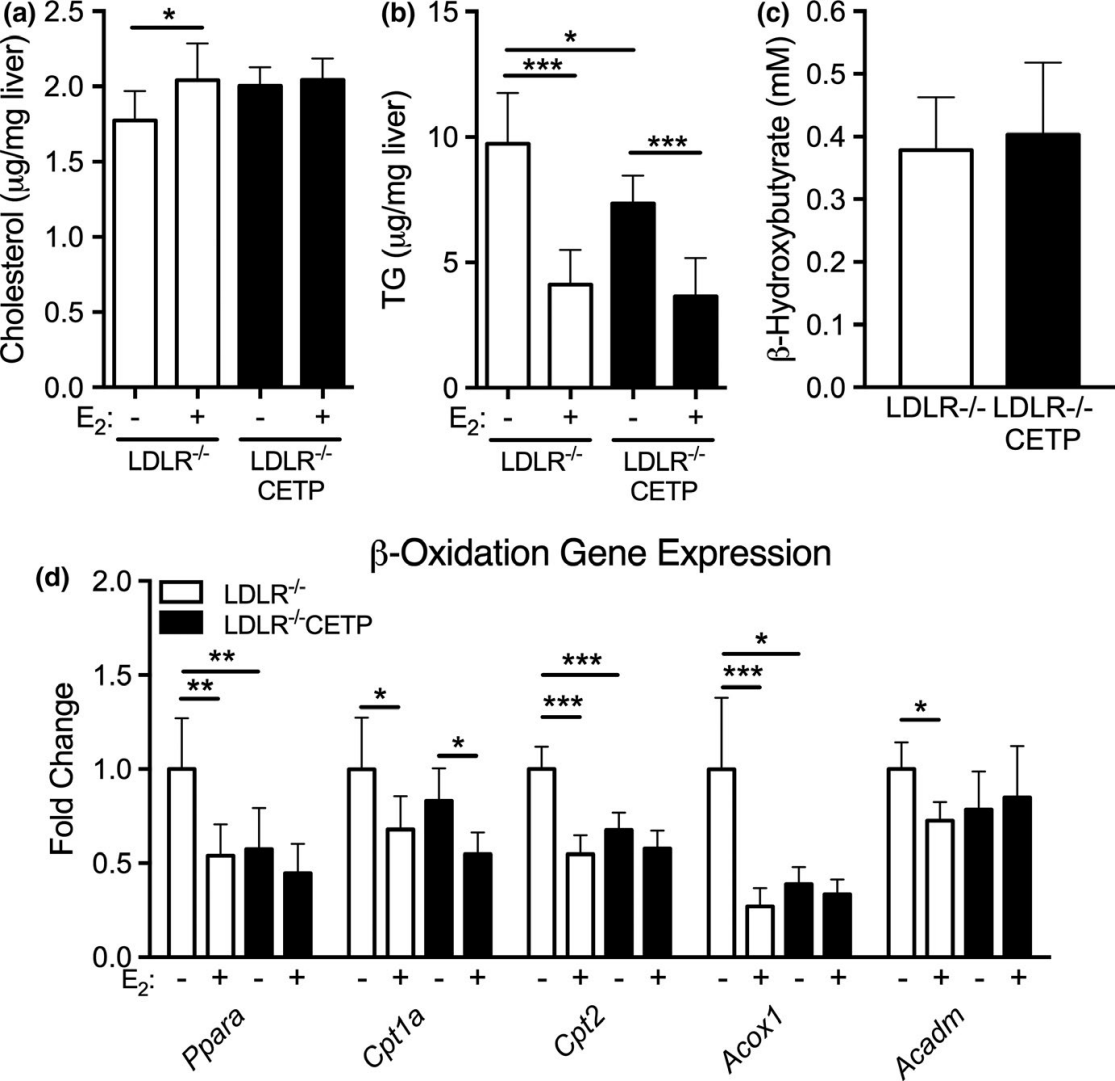
Effect of cholesteryl ester transfer protein CETP on liver lipid content and liver β‐oxidation in females lacking low‐density lipoprotein receptor LDLR. Ad‐lib fed female LDLR^−/−^CETP and LDLR^−/−^ littermates were given sesame oil (veh) or estrogen (E_2_) and were sacrificed 24 h later between 8 and 11 am. (a, b) Liver cholesterol content (a) and liver TG content (b) in LDLR^−/−^CETP and LDLR^−/−^ treated with veh or E_2_. (*n* = 7–8/group, *p < 0.05, ***p < 0.001, one‐way ANOVA with post hoc comparison). (c) Plasma β‐hydroxybutyrate levels were measured in 18 h overnight fasted LDLR^−/−^ and LDLR^−/−^ CETP females (*n* = 6–8/group, *t*‐test). (d) Liver mRNA levels of genes involved in β‐oxidation was measured in liver from ad‐lib fed females treated with veh or E_2_ 24 h prior to study (*n* = 7–8/group, *p < 0.05, **p < 0.01, ***p < 0.001, one‐way ANOVA with post hoc comparison)

We previously showed that CETP expression in females reduced liver steatosis by enhancing liver β‐oxidation (Palmisano et al., 2016). We next determined if CETP was able to alter liver TG content in females in the absence of LDLR. CETP did not alter liver cholesterol content with LDLR deletion (Figure 4a). Estrogen treatment modestly increased liver cholesterol content in LDLR^−/−^ females, but not in LDLR^−/−^CETP females (Figure 4a). In the absence of LDLR, CETP expression reduced liver TG content by 25% relative to control females (7.35 ± 1.11 vs 9.73 ± 2.02 µg/mg liver, LDLR^−/−^ CETP veh vs. LDLR^−/−^ veh, *p* < 0.05, Figure 4b). Estrogen treatment reduced liver TG content to a similar level in both LDLR^−/−^ and LDLR^−/−^CETP females (Figure 4b). Previously, we demonstrated that CETP reduced liver TG content by ~60% relative to WT controls (Palmisano et al., 2016). Thus, CETP lowers liver TG content despite the absence of LDLR, but perhaps to a lesser degree than mice with intact LDLR.

We previously demonstrated that CETP reduced liver TG content by increasing liver β‐oxidation gene expression and activity (Palmisano et al., 2016). Since the deletion of LDLR appeared to lessen the effect of CETP on lowering liver TG content, we next sought to determine if LDLR was required for CETP to enhance liver β‐oxidation targets. To determine this, we measured plasma β‐hydroxybutyrate levels in fasted mice, a total body measure of β‐oxidation activity, and liver mRNA levels of β‐oxidation target genes. In the absence of LDLR, CETP failed to increase fasting plasma β‐hydroxybutyrate (Figure 4c). Additionally, in the absence of LDLR, CETP failed to increase liver mRNA levels of β‐oxidation targets (Figure 4d). In fact, in the absence of LDLR, CETP reduced liver mRNA levels of β‐oxidation targets *Ppara*,*Cpt2*, and *Acox1* (Figure 4d). Estrogen reduced mRNA expression of β‐oxidation target genes (*Ppara*,*Cpt2*,*Acox1*,*Acadm*) in LDLR^−/−^ females, but not in LDLR^−/−^CETP females (Figure 4d). Taken together, these data indicate that LDLR is required for CETP to enhance liver β‐oxidation, but not required for CETP to reduce liver TG content, although the effect of CETP on liver TG content appears diminished in the absence of LDLR. In total, these results demonstrate that LDLR may be an important factor for the ability of CETP to alter plasma and liver TG metabolism in both males and females.

## 4 DISCUSSION

Mechanisms explaining sex differences in lipid metabolism remain poorly understood but are important for understanding the risk of cardiovascular disease in men and women. CETP, although known for its effects on plasma HDL cholesterol lowering, alters TG metabolism in both males and females (Palmisano et al., 2016, 2020). The aim of this study was to determine a unifying mechanism explaining how CETP alters TG metabolism in both males and females. LDLR has established roles in both TG clearance and TG production (Coenen et al., 2007; Ishibashi et al., 1996; Teusink et al., 2001), representing a unique target that may underlie the differing effects of CETP on TG metabolism in males and females. In this study, we demonstrate that LDLR was required for CETP to alter TG metabolism in both males and females. In males, LDLR was required for CETP to raise plasma TGs and impair postprandial TG clearance. In females, LDLR was required for CETP to raise plasma TGs, increase TG production, and increase expression and activity of genes involved in VLDL synthesis and assembly in response to estrogen. Additionally, LDLR was required for CETP to enhance liver β‐oxidation gene expression, but LDLR was only partially required for CETP to reduce liver TG content. Taken together, these data demonstrate that LDLR is required for CETP‐mediated changes in TG metabolism in both males and females.

These data support a role for LDLR in CETP‐mediated regulation of TG metabolism, perhaps beyond the conventional functions of LDLR. LDLR has been previously shown to regulate VLDL synthesis and TG clearance in humans and animal models (Bilheimer et al., 1979; Coenen et al., 2007; Cummings, 1995; de Faria et al., 1996; Horton et al., 1999; Ishibashi et al., 1996; James et al., 1989; Millar et al., 2002; Teusink et al., 2001; Tremblay et al., 2004; Yamamoto et al., 2017; Zulewski et al., 1998). LDLR reduces VLDL production through postendoplasmic reticulum (ER) degradation of apoB and immediate particle reuptake on the cell surface (Blasiole et al., 2008). This model, however, is inadequate to explain how LDLR is required for CETP to regulate expression of β‐oxidation and VLDL target gene expression with estrogen treatment (Palmisano et al., 2016). If LDLR only functioned in post‐ER VLDL processing, the deletion of LDLR would not be required for CETP to regulate PDI activity or estrogen regulation of gene expression. Yet, our data demonstrate that LDLR is required for CETP‐mediated changes in gene expression and PDI protein activity in response to estrogen. Therefore, LDLR likely functions upstream in CETP‐mediated regulation of gene expression in response to estrogen. Thus, LDLR may have additional roles in TG metabolism beyond direct binding and clearance of TG‐rich lipoproteins from the cell surface.

In addition to demonstrating a broader function for LDLR in the context of CETP, our data also suggest a broader role for LDLR in estrogen regulation of gene expression. Our data demonstrate that with LDLR deletion, estrogen regulates genes involved in VLDL synthesis (*Apob*,*Mttp*,*Pdia3*,*Arf1*,*Sort1*, Figure 3d) and β‐oxidation (*Ppara*,*Cpt2*,*Acox1*,*Acadm*, Figure 4d). However, under similar experimental conditions in females of similar genetic makeup (C57Bl/6), we previously showed that estrogen did not regulate expression of *Apob*,*Mttp*,*Arf1*,*Sort1*,*Ppara*,*Acox1*, or *Acadm* in WT females (Palmisano et al., 2016). Thus, LDLR deletion generated a gain of function response to estrogen in the liver. Previous work has demonstrated that LDLR deletion impairs fertility in female mice, but this was due to reduced ovarian follicular function (Guo et al., 2015). This is the first evidence, to our knowledge, that LDLR has a role in sex hormone signaling in the liver. The physiologic impact of this finding warrants further investigation, but is beyond the aim of the current study, which was to understand how CETP altered TG metabolism in male and female mice.

Deletion of LDLR in mice is one of the most common methods to study dyslipidemia and atherosclerosis in animal models. This study utilized LDLR knockout mice to determine if LDLR was required for CETP‐mediated alterations in TG metabolism. An alternative hypothesis to the model presented here is that deletion of LDLR is maximally deleterious to mouse models of lipid metabolism, which precludes further worsening of dyslipidemia by CETP and other targets governing TG metabolism. However, several studies support that further manipulation in the setting of LDLR knockout can further worsen the dyslipidemia seen in LDLR knockout mice (Coenen et al., 2007; Fuller et al., 2014; Hasty et al., 2001; Ishibashi et al., 1994; Karasawa et al., 2011; Rensing et al., 2014). Additionally, several studies demonstrate that dyslipidemia can be improved in the setting of LDLR knockout (Gordts et al., 2016; Saraswathi et al., 2007). It is unlikely, therefore, that the failure of CETP to alter TG metabolism in the absence of LDLR was because mouse physiology cannot accommodate further worsening of dyslipidemia beyond that seen with deletion of LDLR.

An unanswered question until this point was how CETP, a secreted plasma protein, alters plasma TG levels, intracellular TG metabolism, and sex hormone receptor function in the liver. Data presented here shed light on this question. Two possible hypotheses could connect CETP function to LDLR regulation of TG metabolism and nuclear receptor function. One hypothesis is that CETP alters intracellular delivery of signaling molecules to pathways regulating TG metabolism and nuclear receptors. A second hypothesis is that plasma CETP alters the route by which lipids enter cells, with subsequent differences in TG metabolism and nuclear receptor signaling. Data presented here could support the first hypothesis if intracellular CETP alters lipid delivery from LDLR‐derived endosomes to TG metabolic and nuclear receptor targets. *In vitro* work supports a role for intracellular CETP in regulating TG metabolism and LDL uptake in adipocytes and liposarcoma cells (Greene et al., 2015; Izem et al., 2015; Izem & Morton, 2007). The second hypothesis, however, may more easily explain the broad functional impacts of LDLR deletion on CETP function in TG metabolism. These hypotheses are not mutually exclusive and may occur concurrently. Nonetheless, these data support a novel role of LDLR in CETP‐mediated alterations in liver TG metabolism in both males and females. Future work will be aimed at delineating the molecular intermediates of this novel CETP‐LDLR pathway to TG metabolic pathways and nuclear receptor signaling. Additionally, defining the role of LDLR in sex hormone regulation of TG metabolic gene expression may reveal novel regulatory pathways in TG metabolism.

## CONFLICT OF INTEREST

J.C.N. is currently a Novo Nordisk Inc. employee but was at Vanderbilt during the time the studies were performed. All other authors declare that they have no conflicts of interest.

## AUTHOR CONTRIBUTIONS

B.P and J.S. contributed to the formation of the overall concept. B.P, S.Y, J.N, L.Z, and T.L. performed the experiments, analyzed the results, and made the figures. B.P wrote the original manuscript. All authors had complete access to all the study data, contributed to drafting and critically revising the manuscript. All authors approved the final version of the manuscript and take responsibility for the integrity of the data and the accuracy of the data analysis.
